# Supplementary figures and images for: Structural roles of Ump1 and β-subunit propeptides in proteasome biogenesis
Source: Life Sci Alliance. 2024 Sep 11;7(11):e202402865. doi: 10.26508/lsa.202402865 (PMC11391049; doi:10.26508/lsa.202402865)

Figure S1A

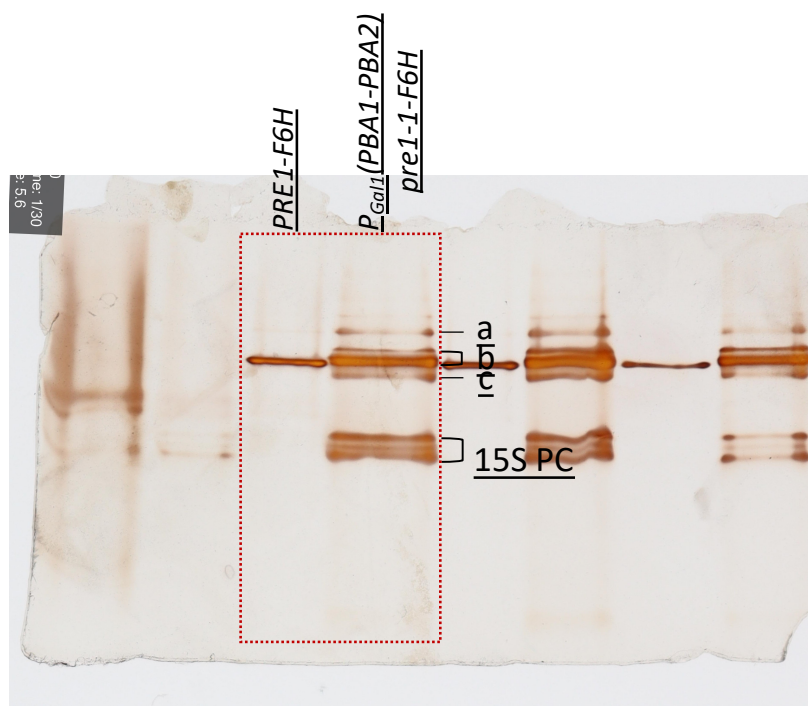

Supplement: Supplementary file 1 [file LSA-2024-02865_SdataFS1.pdf]

Figure S3A

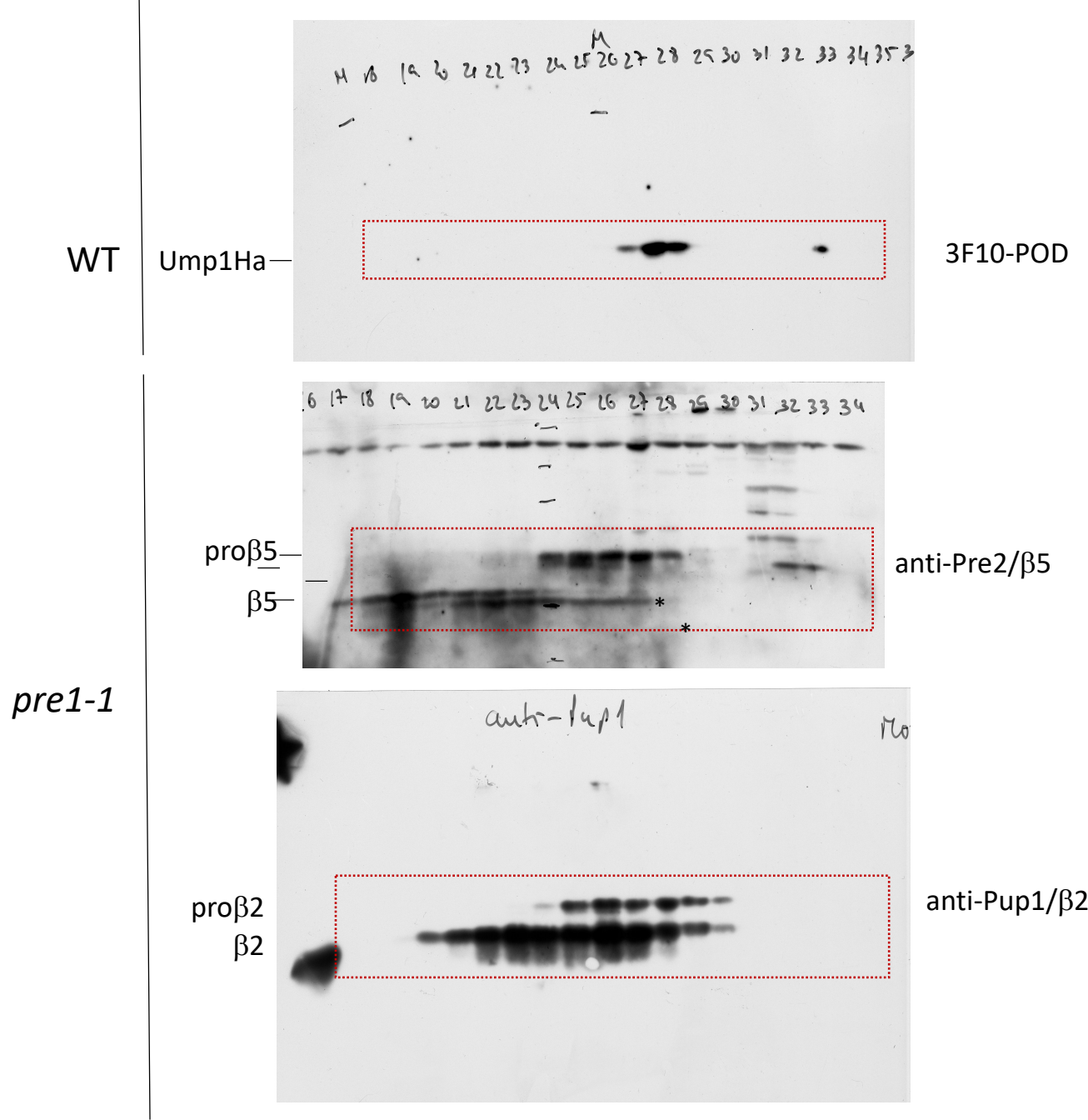

Figure S3B

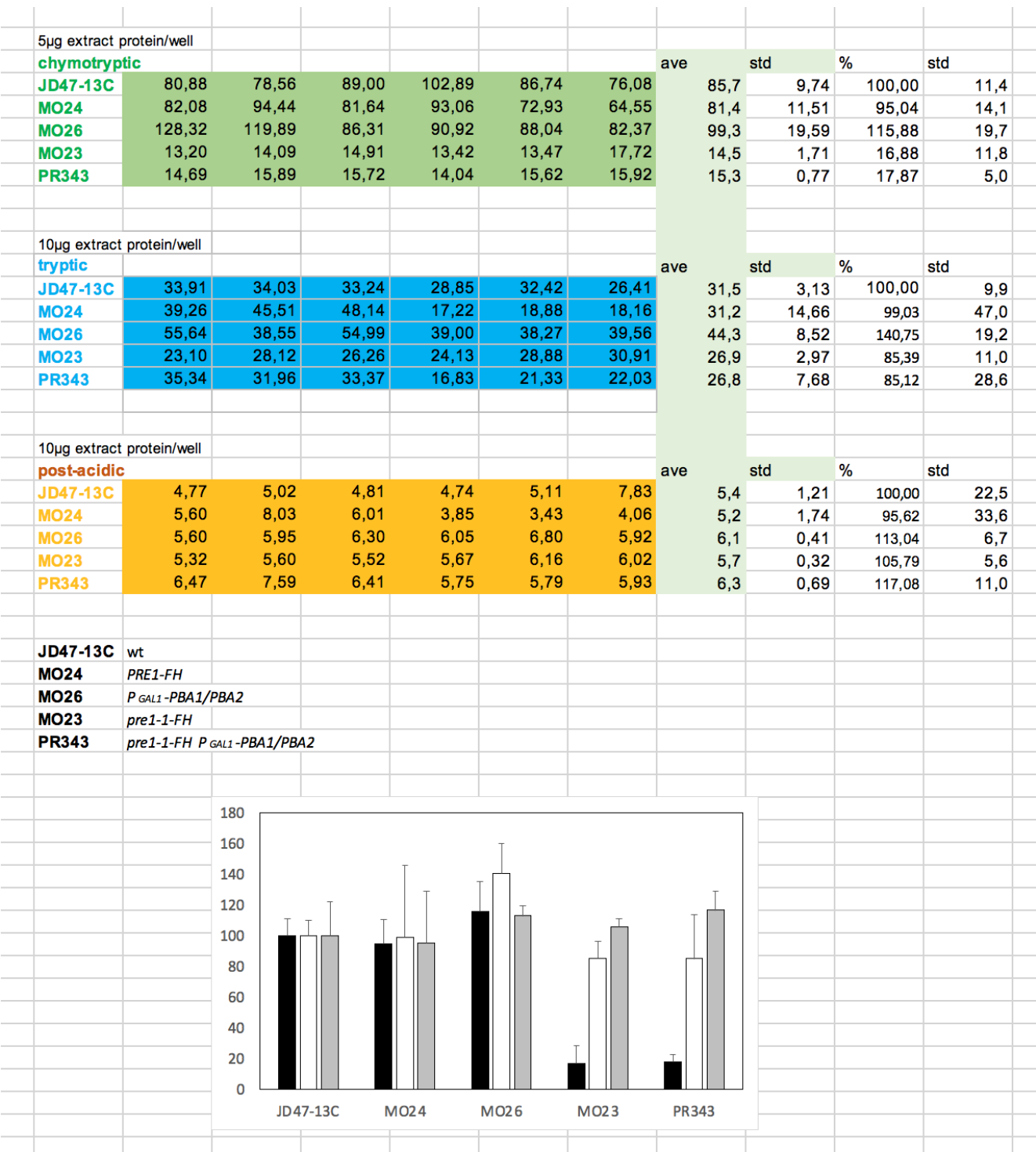

Supplement: Supplementary file 2 [file LSA-2024-02865_SdataFS3.pdf]

Figure S14

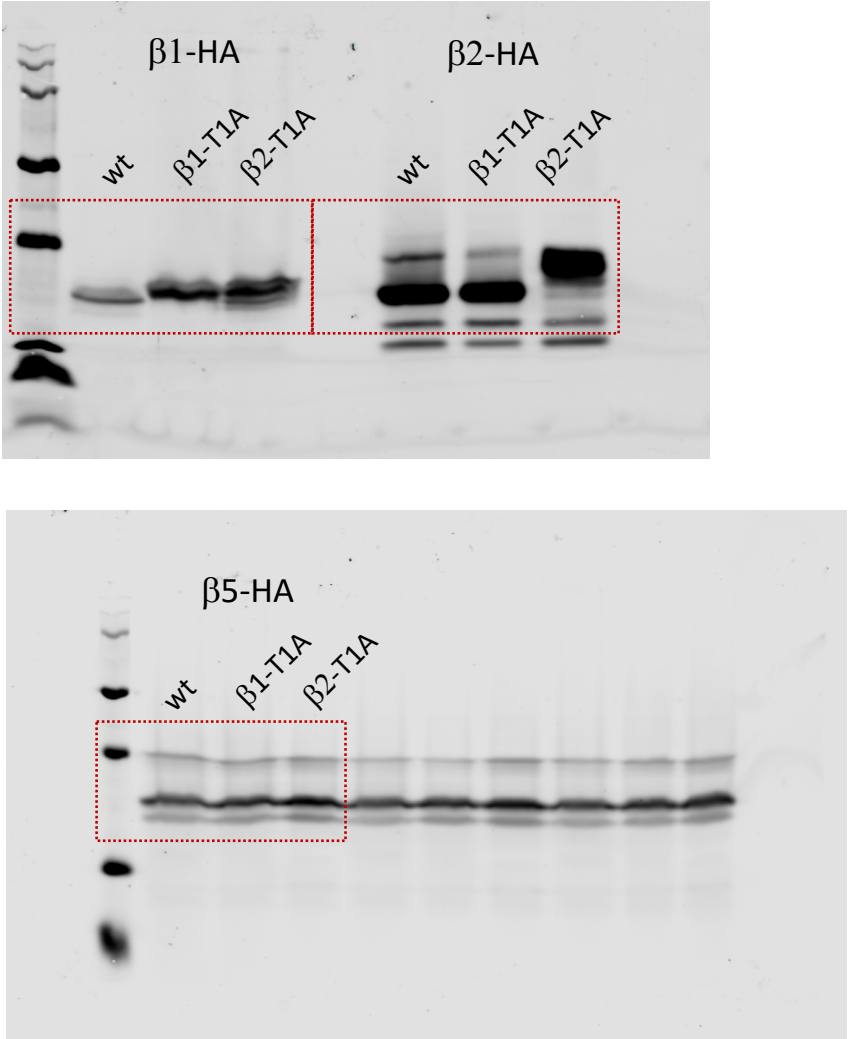

Anti-HA blots

Supplement: Supplementary file 5 [file LSA-2024-02865_SdataFS14.pdf]
