# Supplementary material for: Structural roles of Ump1 and β-subunit propeptides in proteasome biogenesis
Source: Life Sci Alliance. 2024 Sep 11;7(11):e202402865. doi: 10.26508/lsa.202402865 (PMC11391049; doi:10.26508/lsa.202402865)
Supplement: Supplementary file 4 [file LSA-2024-02865_TableS1.docx]

|  | late-PC | | | *^pre1‑1^* CP | ^pre1-1^CP-Blm10 |
| --- | --- | --- | --- | --- | --- |
|  | Symmetrized | State1 | State 2 |  |  |
| EMDB | 19523 | 19527 | 19528 | 19529 | 51221 |
| PDB | 8RVL | 8RVO | 8RVP | 8RVQ | 9GBK |
| **Data collection** | | | | | |
| Magnification | 105 000 | | | | |
| Voltage [kV] | 300 | | | | |
| Electron exposure [e^-^/Å^2^] | 44.00 | | | | |
| Defocus range [µm] | 0.6 ‑ 2.0 | | | | |
| Pixel size [Å] | 0.834 | | | | |
| **Processing** | | | | | |
| Initial particle images [no.] | 394,211 | | | 457,964 | 178,981 |
| Final particle images [no.] | 233,748 | 53,919 | 169,752 | 341,154 | 129,737 |
| Symmetry imposed | C2 | C1 | C1 | C2 | C1 |
| Map resolution [Å] | 2.14 | 2.69 | 2.28 | 2.02 | 2.39 |
| FSC threshold | 0.143 | 0.143 | 0.143 | 0.143 | 0.143 |
| **Refinement** | | | | | |
| Initial model used [PDB code] | *de novo* | *de novo* | *de novo* | *de novo* | *de novo* |
| Model resolution [Å] | 2.14 | 2.69 | 2.28 | 2.02 | 2.39 |
| FSC threshold | 0.143 | 0.143 | 0.143 | 0.143 | 0.143 |
| Map sharpening B‑factor [Å^2^] | 44.6 | 32.2 | 32.2 | 33.7 | 42.7 |
| Model composition | | | | | |
| Non-hydrogen atoms | 62,379 | 61,792 | 61,984 | 51,191 | 64,984 |
| Protein residues | 7,626 | 7,627 | 7,620 | 6,270 | 7,941 |
| Ligands | 0 | 0 | 0 | 0 | 0 |
| R. m. s. deviations | | | | | |
| Bond lengths [Å] | 0.002 | 0.007 | 0.003 | 0.002 | 0.002 |
| Bond angles [°] | 0.458 | 0.483 | 0.523 | 0.491 | 0.427 |
| Validation | | | | | |
| MolProbity score | 1.26 | 1.48 | 1.41 | 1.52 | 1.86 |
| Clashscore | 4.27 | 5.90 | 4.84 | 4.99 | 6.21 |
| Poor rotamers [%] | 1.17 | 1.25 | 1.31 | 1.49 | 2.36 |
| Ramachandran plot | | | | | |
| Favored [%] | 98.10 | 97.59 | 97.70 | 97.34 | 96.42 |
| Allowed [%] | 1.86 | 2.37 | 2.29 | 2.62 | 3.49 |
| Disallowed [%] | 0.04 | 0.04 | 0.01 | 0.03 | 0.09 |
